# The Quest for Antibodies and Other Acquired Immune Receptors: A Historical Perspective

**DOI:** 10.1111/iji.12712

**Published:** 2025-05-08

**Authors:** Andrea De Lerma Barbaro, Sahar Balkhi, Stefano Giovannardi, Alberto Vianelli, Domenico Ribatti, Lorenzo Mortara

**Affiliations:** ^1^ Department of Biotechnology and Life Sciences Laboratory of Comparative Physiopathology University of Insubria Varese Italy; ^2^ Department of Biotechnology and Life Sciences Immunology and General Pathology Laboratory University of Insubria Varese Italy; ^3^ Centre for Neuroscience University of Insubria Varese Italy; ^4^ Department of Human Sciences and Sciences of the Innovation for the Territory University of Insubria Varese Italy; ^5^ Department of Translational Biomedicine and Neurosciences University of Bari Medical School Bari Italy

**Keywords:** acquired immune receptors, history of biological sciences, non‐model vertebrates, non‐vertebrate adaptive immunity

## Abstract

The diversity of antibody molecules has for decades been an unsolved enigma that has attracted wide interest among biologists. Parallel to the accumulation of experimental evidence, progress in antibody research was also driven by the theoretical debate that played a particularly prominent role, at least until the entry of molecular biology into this field of investigation. Several publications have examined this topic from a historical perspective. In this article, we aim to examine the history of research into the mechanisms underlying antibody diversity from a partly new standpoint. In jawed vertebrates (gnathostomes), progressively more distant on the evolutionary scale from humans and mice—in non‐model mammals, birds, amphibians, bony and cartilaginous fish—certain mechanisms for the diversity of acquired immunity receptors (B‐cell receptors [BCR]/immunoglobulins [Ig] and T‐cell receptors [TCR]) have been described that are quite unexpected on the basis of what has emerged from biomedical immunology studies. What is more, in *Agnatha* vertebrates, in several invertebrate phyla and even in bacteria, forms of adaptive immunity have been discovered, based on the ability to finely tune the host defence response to the infectious threats. These defence systems show some similarities with the acquired immunity of jawed vertebrates, although they are based on mechanisms and receptors totally different from BCR/Ig and TCR. Therefore, our aim is to investigate how the theoretical debate on antibody diversity, which developed in the 20th century, partly anticipated some of the central themes in the current research on adaptive immunity systems discovered in the previously mentioned non‐model systems. With this aim, we have reformulated, in the language of modern biology, some of the hypotheses advanced in the first decades of antibody diversity research.

## 1 Introduction

The immune system of jawed vertebrates (gnathostomes) distinguishes between self and non‐self primarily through two categories of receptors. Innate immune receptors, encoded directly in the germline and often referred to as pattern recognition receptors (PRRs), are ‘ready‐made’ to bind and recognize pathogen‐associated molecular patterns (PAMPs) (Janeway 1989).

Conversely, acquired immune receptors, namely, B‐cell receptors (BCRs)/immunoglobulins (Ig) and T‐cell receptors (TCRs), require a process of gene rearrangement to become functional. These receptors bind and recognize specific antigens. For decades, antibodies were the only known receptors of acquired immunity, and their remarkable ability to recognize an almost limitless array of antigens has puzzled generations of immunologists and biologists. This puzzle is often referred to as the ‘generator/generation of diversity’ (GOD) paradox, a term coined by Dick Gershon (Golub 1982).

In this review article, we conduct a historical analysis of experimental and theoretical investigations into the biological paradox of antibody diversity, examining the contributions of key scientists who played pivotal roles in this field. We will focus primarily on the theoretical debate surrounding the mechanisms of antibody diversity, which laid the foundation for subsequent experimental investigations. Our aim is to discuss the hypotheses proposed by early researchers and, at times, to reinterpret their ideas using the language of modern biology. We selectively highlight the theoretical contributions that have had the most significant and lasting impact.

Starting in 1957, with MacFarlane Burnet's theoretical study on clonal selection, awareness and interest in the GOD paradox grew within the scientific community. This interest peaked with Tonegawa's (Hozumi and Tonegawa 1976) experimental demonstration of *Ig* gene rearrangement and gradually waned in the following years as advances in molecular biology answered many of these longstanding questions. Since the late 1990s, it can be stated with confidence that, aside from minor details, the biology of acquired immune receptors has been thoroughly elucidated in the most extensively studied organisms, namely mice and humans, which are therefore referred to as model mammals.

The primary objective of this article is to emphasize that research on the GOD mechanisms has extended beyond model mammals since the 1960s, encompassing non‐model mammals and, more recently, organisms further down the evolutionary scale, including birds, amphibians, bony fish and cartilaginous fish. This research has uncovered novel and sometimes unexpected ‘solutions’ to the GOD conundrum, some of which were anticipated in the theoretical debates of the 1960s. Moreover, these comparative studies have enhanced our understanding of the distinct functional roles of BCRs/Ig and TCRs, even within model mammalian systems.

### 1.1 Principal Milestones From P. Ehrlich (1900) to S. Tonegawa (1976)

In this section, we outline the main landmarks in research aimed at understanding antibody responses. We will focus primarily on the intense theoretical debate in Box S1 that continued until 1976, when molecular biology entered the field, allowing for direct analysis and providing informative answers to many questions raised by the paradoxical aspects of antibody responses. Indeed, after 1976, theoretical debate played a relatively minor role.

Given the broad time span under consideration, the following exposition is presented in a linear and simplified format, which may seem somewhat naive from the perspective of a historian of biology or medicine. We focus only on the main theories, based on their prominent discussion in key sources, that reconstruct the history of antibody research (Silverstein 2003a; Hodgkin et al. 2007; Jordan and Baxter 2008; Ribatti 2009).

Additionally, we do not delve into the various authors who have occasionally proposed similar ideas. For a comprehensive treatment of the theoretical debates surrounding antibodies, please refer to the following book (Silverstein 2009).

### 1.2 Dark Ages of Immunology 1890–1957

The discovery of antibodies can be traced back to 1890, when Emil von Behring and Shibasaburo Kitasato published a landmark study showing that transferring serum from animals immunized with heat‐killed diphtheria bacteria to animals infected with virulent diphtheria could improve the course of the disease (Von Behring and Kitasato 1890).

This result, along with others that followed, was interpreted by hypothesizing the appearance in serum of a substance that specifically interacts with toxic molecular components produced by pathogens or with noxious plant chemicals. These substances, capable of attenuating the damaging effects of pathogens and environmental toxins, were initially referred to as antitoxins and later called antibodies (Kantha 1991).

Protagonist of this first phase, particularly in the theoretical debate resulting from the experimental evidence, was Paul Ehrlich (Box S2).

For several years, it seemed plausible that antibodies could recognize a significant, yet limited number of substances associated with germs or other environmental toxins. This idea, consistent with Darwinian theory, suggested that organisms had encountered such substances in their evolutionary past, although this notion was not always clearly articulated. To explain these observations, Paul Ehrlich proposed a series of assumptions summarized in the side‐chain theory (Ehrlich 1900).

The key feature of Ehrlich's model was the existence of a *preexisting* repertoire of antibodies, each specifically attuned to various toxic substances the organism might encounter. According to this theory, antibody‐producing cells have ‘side chains’ on their protoplasm (cell surface) that can specifically interact with toxins.

As a result of the interaction between a toxin and a side chain, the cell would release the side chain as a soluble antibody into the surrounding environment. Subsequently, in a compensatory process, the cell produces and secretes an excess of antibodies with the same specificity.

The understanding of antibody responses shifted dramatically within a few years. Researchers found that even seemingly innocuous proteins, such as chicken egg albumin, and cells like erythrocytes (from different organisms) could stimulate the formation of specific antibodies. This raised a significant question: What is the evolutionary benefit of producing antibodies against these harmless immunogens? Moreover, the repertoire of the immune system appeared even larger when, in 1906, Obermayer and Pick discovered that covalent modifications of proteins could alter their antigenic profiles (Obermayer and Pick 1906). This pioneering observation paved the way for the experiments of Landsteiner and his colleagues (Landsteiner 1947). They demonstrated that antibodies could be raised against almost any synthetic chemical structure (hapten) as long as the hapten was first conjugated to a carrier protein and injected alongside an adjuvant, such as killed tubercle bacilli.

The discovery of an apparently unlimited variety of antibodies led scientists, from the 1920s onwards, to discard the notion that the body contained preexisting information for all potential antibodies. As a result, instructive theories emerged, proposing that the antigen itself supplied all the necessary information to shape the specific antibody, effectively acting as a template. By the 1930s, scientists had identified antibodies as globular proteins, composed of 20 different amino acids in varying sequences. However, it remained unclear whether there was any regularity or specificity in these amino acid arrangements and how such information for protein structure was stored and accessed (Edsall 1979). On these premises, in 1930, a new hypothesis for antibody formation, completely different from Ehrlich's side‐chain theory, was proposed by Breinl and Haurowitz (1930). The antigen was thought to act as a template for antibody synthesis, guiding the addition of amino acids to the nascent antibody protein. In this model, the antigen determines the specificity of the antibody. Ten years later, in 1940, the renowned chemist Linus Pauling significantly modified the Breinl and Haurowitz hypothesis by imagining antibodies as a unique, ‘shapeless’ protein product (Pauling 1940). According to Pauling, the antigen would serve as a template for the final step of protein formation by promoting complementary, mirror‐like folding in the antibody protein.

Although these instructive theories were widely accepted at the time—as they provided a simple explanation for antibody specificity and diversity—they were poorly supported by experimental evidence and failed to account for most functional observations. In particular, the template models proposed by Breinl and Haurowitz, and Pauling allegedly required the antigen to persist throughout the course of antibody formation. This complicates the explanation of the rapid, logarithmic increase in antibody titres during an immune response. Additionally, these theories did not adequately explain immunological memory or self‐tolerance, concepts introduced by Macfarlane Burnet in the early 1940s through theoretical reasoning.

Macfarlane Burnet Box S2, an Australian virologist, subjected these template theories to intense criticism, simultaneously attempting to modify them to better align with functional evidence. Through his theoretical work, Burnet paved the way for new advances in the field.

The event unanimously regarded as a key turning point was Jerne's (1955) article, which explicitly referred to the Ehrlich theory of side chains returning to a selectionist view. According to Jerne's natural‐selection theory, the body synthesizes small amounts of antibodies that are specific to a broad range of antigens. These ‘natural’ antibodies are present in the blood before encountering the antigen. Upon antigen binding, the specific antibody is selected and transported to specialized cells capable of over‐producing antibodies of the same specificity. In Jerne's model, the antigen merely serves as a ‘selective carrier’ for the antibody, guiding the synthesis or modification of RNA specific for the antibody. Jerne also explained self‐tolerance by suggesting that self‐reactive antibodies are sequestered and neutralized through binding to self‐antigens early in development.

Two years later, in 1957, Burnet, explicitly referring to Jerne's contributions, reintroduced Ehrlich's ideas in his Clonal Selection Theory (Burnet 1957). Burnet proposed that antibodies specific to a vast array of antigenic structures, pre‐existing prior to exposure, are expressed as membrane receptors on specialized cells. In contrast to Ehrlich's side‐chain theory, Burnet hypothesized that each cell expresses antibodies specific to a single antigen. As a result, a large number of ‘virgin’ (naive) cells, each specific for a different antigen, pre‐exist the antigenic challenge. Upon contact with the antigen, the cell expressing the specific receptor undergoes activation and clonal expansion, differentiating into antibody‐secreting cells and memory cells. Finally, tolerance to self would arise through ‘clonal abortion’, where self‐reactive cells die before reaching a critical level of maturation.

All hypotheses for antibody diversity proposed between 1900 and 1957 had strengths and weaknesses; indeed, each failed to explain certain fundamental aspects of the paradox. Although this must have been frustrating, it also served as a stimulus for greater curiosity and interest in the scientific community. The side‐chain hypothesis assumed that the information contained in antibodies was pre‐existing in the organism, what we would now say is sorted (encoded) in the genome. In contrast, instructive hypotheses proposed that the information for antibody specificity came entirely from the structure of the antigen. Side‐chain theory explained several functional aspects of antibody responses but failed to explain diversity and specificity. Instructive theories, although useful for explaining specificity and the vast size of the antibody repertoire, struggled to account for the rapid antibody response, memory and self‐tolerance.

### 1.3 Modern Immunology (1957–1976)

Burnet's hypothesis was quickly embraced by the scientific community, marking the beginning of modern immunology, which was characterized by a renewed focus on the functional aspects of immune responses. In the early 1960s, the study of cells involved in immune responses—both in vivo and ex vivo—was greatly advanced by the discovery of T and B lymphocytes (Nagy 2013).

Burnet's hypothesis had the advantage of suggesting numerous experimental tests that could verify or falsify the theory, some of which were within reach of the technological capabilities of the 1960s. First, Nossal and Lederberg (1958) provided evidence supporting the idea that antibody‐secreting plasma cell clones produce no more than one antibody species. Additionally, in 1961, Goran Moller, using fluorescein labelled anti‐antibodies, provided the first evidence that B lymphocytes in peripheral blood express Ig on their surface (Moticka 2015). Furthermore, injection of radiolabelled antigens resulted in clonal deletion; in other words, the remaining in vivo cell population could no longer respond to the same ‘cold’ unlabelled antigen (Ada and Byrt 1969). Finally, one of the most compelling pieces of evidence for the clonal selection theory came in the 1970s with the technique introduced by Köhler and Milstein (1976) to generate monoclonal antibodies (Nagy 2013; Ribatti 2014). It is important to note that in its original formulation, Burnet's clonal selection theory did not specify the mechanism responsible for generating antibody diversity.

Burnet's model sparked an intense debate, particularly regarding the genetic implications of antibody diversity. J. Lederberg (1959), a distinguished geneticist and Nobel Laureate, suggested that antibody diversity must depend on a high somatic mutation rate in the Ig encoding gene(s). On the other hand, Talmage argued statistically in favour of a large antibody repertoire that was already fully encoded in the germline, meaning that it existed in the genome at birth. Indeed, at that time, it was well understood that antibody responses were characterized by cross‐reactivity: Antibodies could interact with high affinity with the antigen they were generated against and with lower but still significant affinity with other, structurally related antigens. Therefore, Talmage hypothesized that, because multiple antibodies could coexist in a single humoral response, the combination of these antibodies could produce a seemingly unlimited number of responses along a cross‐reagent *continuum* (Talmage 1959). According to this view, a few thousand distinct, germline‐encoded antibodies can cover an unlimited antigenic universe.

Thus, a division was emerging between proponents of the somatic hypothesis (Lederberg and Burnet) and those who believed that the Ig repertoire was germline‐encoded (Talmage). Proponents of the germline hypothesis argued that antibody diversity was generated by a large pool of genes encoding Igs, which existed in the genome as distinct genes (the multigene hypothesis). In contrast, proponents of the somatic hypothesis believed that only a few genes were involved, but these genes underwent somatic mutations (the paucigene hypothesis) to generate diversity (Silverstein 2003a). When the basic structure of Ig was determined in the mid‐1960s, the so‐called p × q hypothesis was proposed. This hypothesis suggested that, for example, 3000 light chains and 3000 heavy chains‐encoded germline by ‘only’ 6000 genes in the germline could give rise to approximately 10^7^ different antibodies (Hood and Talmage 1970). The p × q hypothesis is a radical form of multigene theory.

The peak of the theoretical debate came in 1965 when Dreyer and Bennett (1965) published a hypothesis that had a profound impact on the scientific community and later proved to be fundamentally correct. Drawing on sequence data from Bence‐Jones proteins, which showed that antibody light chains consist of two portions—variable at the amino‐terminal and constant at the carboxy‐terminal—Dreyer and Bennet hypothesized that the *Ig* gene is split into two segments in the germline. These segments are later joined in B cells to form a single functional gene unit. This hypothesis represented a synthesis of germline and somatic theories. Variability would be encoded in the germline, but the functional gene unit would be formed through a somatic process, later termed somatic recombination—distinct from the recombination that occurs during gametogenesis.

The debate between germinalists and somaticists gave rise to polarized arguments regarding antibody diversity, each carrying distinct evolutionary and developmental implications (Kindt and Capra 1984; Silverstein 2003a). According to the germinalist view, the Ig repertoire, already present at birth, would have originated in the evolutionary past of the species, under the selective pressure of the arms race between the host and its pathogens. In contrast, according to somaticists, the Ig repertoire would originate during ontogenetic development.

In several vertebrates, evidence indicates that the organism manifests early antibody responsiveness towards a broad antigenic repertoire. This state of immunocompetence at an early stage characterized by low lymphocyte numbers (the so‐called tadpole paradox) (Langman and Cohn 1987) was interpreted as an argument against the somaticist hypothesis. Indeed, ontogenetic data were generally used to support the germinalist hypothesis although it has been shown that even small numbers of lymphocytes in early ontogenetic development are compatible with the function of acquired immunity (Du Pasquier 1970). On the other side, one of the most telling arguments used by supporters of the somaticist model against germinalist pertains to Darwinian evolution. The large gene pool hypothesized by germinalists should be maintained from generation to generation even if each individual organism, during its lifetime, will make use of only a small fraction of the entire repertoire. Somaticist proponents claimed that, in the absence of positive selective pressures, it would not take long for unused genes to lose their sequence identity (Silverstein 2003a) (see Section 4 for further discussion of this topic).

From 1976 onwards, speculation had fewer places since the emerging molecular biology of the gene allowed more direct testing of the mechanisms underlying antibody diversity. In that year, Tonegawa's work provided a significant breakthrough. By comparing B lymphocyte DNA with germline DNA after digestion with restriction enzymes, he demonstrated that the gene encoding the Ig light chain undergoes somatic recombination during B cell differentiation. This discovery substantially confirmed the hypothesis proposed by Dreyer and Bennett. In essence, it was shown that *IgL* gene organization differs between antibody‐expressing cells (B lymphocytes and plasma cells) and other somatic or embryonic cell lineages (Tonegawa 1988). Subsequent sequencing of *Ig* genes revealed another surprise: The segment coding for the variable amino‐terminal domain of the light chain was shorter than expected. The missing 39 nucleotides, coding for the last 13 amino acids of the light chain, were located about 2.5 kb upstream of the DNA encoding the constant region of the light chain. This upstream segment was named ‘joint *J*’ and represented a novel gene segment family. So, the major revelation was that the *V* domain of the light chain is encoded not by one but by two separate segments, referred to as *V* and *J* (Murphy 2022). A similar work was conducted shortly thereafter on the heavy chain; again it was shown that the variable region is encoded by three gene segments: *V* (variable), *D* (diversity) and *J* (joining) (Murphy 2022). These segments are spaced apart in the genome but are made contiguous by somatic recombination, significantly increasing the combinatorial diversity at the *Ig* loci. Therefore, understanding the fine structure of *Ig* genes helped resolve many of the mysteries surrounding antibody diversity. However, further progress would depend on characterizing the enzymatic machinery mediating somatic recombination and antibody diversification (Box S1).

In line with what commonly occurs in other areas of scientific inquiry, the resolution of the debate between somaticists and germinalists revealed that the core concepts of both sides were essentially valid. Advocates of the paucigene hypothesis had to concede that antibody diversity is, at least in part, derived from a substantial repertoire of gene fragments, as proposed by the germinalist hypothesis. They also found support for their views in the discovery that diversity within the CDR3 region—formed at the junction point during somatic recombination—arises largely from the random insertion of amino acids into the polypeptide chain. Furthermore, the later discovery of somatic hypermutation (SHM) (Box S1), which accounts for affinity maturation in antibody responses, provided additional validation for the somaticist perspective. Box S3 summarizes the current view of the mechanisms that generate antibody and TCR diversity and plasticity in both mice and humans.

## 2 Acquired Receptors in Vertebrates

The study of antibodies from a comparative physiology perspective across various vertebrate taxa began prematurely, at a time when the mechanisms underlying antibody diversity were still widely unknown. Notably, Ig molecules in cartilaginous fish, the group of vertebrates most evolutionarily distant from mammals, were identified as early as the mid‐1960s (Marchalonis and Edelman 1965).

Characterization of TCRs in non‐model vertebrate species came later, as these receptors of acquired immunity are exclusively expressed on the cell surface, making their study more laborious, even in mice and humans. Finally, with the advent of the necessary molecular biology tools in the late 1970s, the genes encoding BCR/Ig and TCRs have gradually been characterized across various vertebrate classes. The comparative study of acquired immunity receptors has progressed in parallel with the study of the molecular, cellular and tissue components that collectively contribute to B and T responses (Box S1).

Genome analysis across various classes of living vertebrates has revealed that genes encoding antibodies are organized in two distinct configurations. The translocon configuration, found in both *IgH* and *IgL* genes in tetrapods and in the *IgH* gene of bony fish, features multiple *V*, *D* and *J* segments located upstream of the *Ig C* domains for all isotypes. In contrast, cartilaginous fish Ig genes and bony fish *IgL* genes are organized into multiple clusters (miniloci), where a single *V* segment, one or more *D* segments and a single *J* segment are positioned upstream of the *C* region exons of a single isotype (Flajnik 2002).

A primary focus has been to assess whether antibody responses in the various vertebrate classes exhibit functional attributes equivalent to those found in model mammals, such as clonality, affinity maturation, isotype switching, memory and tolerance to self (de los Rios et al. 2015). The answer to this question is generally affirmative, although early data suggested, for example, that affinity maturation did not occur in lower vertebrates. The current view is that affinity maturation also exists in ectodermic vertebrates, although the fold increase is lower than in mammals; moreover, these organisms lack germinal centres (Muthupandian et al. 2021). Furthermore, primary and secondary lymphoid organs have been identified in cartilaginous fish for more than 20 years, and there is information about ontogenetic development and functional compartmentalization in these tissues (Rumfelt et al. 2002). Nevertheless, there still is much to be determined regarding the anatomy and physiology of these processes in lower vertebrates.

The overall diversity of Ig molecules depends on several factors, including the germline organization of Ig coding genes and the mechanisms for recombinatorial, primary, antigen‐independent repertoire generation, as well as post‐recombinatorial, secondary, antigen‐dependent repertoire formation. Different vertebrate taxa have evolved distinct mechanisms to generate antibody diversity (Flajnik 2002).

An exhaustive review of the mechanisms of antibody diversity in all vertebrates analysed so far is beyond the scope of this article; therefore, we focus on representative gut‐associated lymphoid tissue (GALT) species and cartilaginous fish (Table 1). The former, while closely related taxonomically to biomedical model mammals, exhibit very different mechanisms, whereas the latter, as the oldest extant vertebrates, offer a potential view of the ancestral acquired immune system.

**TABLE 1:** Relative contribution of the various mechanisms of antibody diversity in representative vertebrate species.

		Primary lymphoid organs antigen‐independent				Secondary lymphoid organs antigen‐dependent	
		Combinational diversity RAG1/RAG2	Junctional diversity RAG1/RAG2, TdT	Somatic hypermutation AID	Gene conversion AID	Somatic hypermutation AID	Gene conversion AID
PR species	Mice/Humans	++	++			++	
GALT species	Chicken		+		++	++	++
	Rabbit	+	+	++	++	++	++
	Shark	Different miniloci involved	++			++	

*Note*: For the meaning of PR and gut‐associated lymphoid tissue (GALT) species, refer to the main text. The anatomical‐functional context and characterizing/signature enzyme activities are also indicated. ‘+’ indicates low/medium relevance; ‘++’ indicates high relevance; blank indicates no role.

Most mammals, and perhaps all birds, generate their primary Ig repertoire not by the combinatorial joining of many *V*, *D* and *J* segments, but rather by diversifying rearranged *VDJ* and *VJ* genes through SHM and/or gene conversion in GALT (Flajnik 2002). Model organisms that belong to the GALT species category include the chicken, rabbit and sheep; the alternative mechanisms for generating Ig diversity in these species would likely never have been discovered through studies of mice and humans. The chicken is particularly illustrative due to its simplicity (Flajnik 2002). In this species, for both *IgH* and *IgL* genes, there is a single functional *V* fragment that rearranges to form a *V(D)J* unit, which then serves as a substrate for subsequent gene conversion events leading to the primary antibody repertoire. Gene conversion is a mechanism that transfers stretches of nucleotides from donor *V* pseudogenes, located upstream, to the rearranged *V(D)J* segment. In this unidirectional gene transfer process, the donor sequences remain unaltered (Flajnik 2002; Lanning and Knight 2015). The key enzyme driving gene conversion is AID, which is also the molecular signature for SHM and isotype switching. As a result, in chickens, there is no combinatorial diversity and little junctional diversity; the primary repertoire is mostly due to gene conversion. Notably, in this species, Ig diversity arises almost exclusively from the random dynamics hypothesized by somaticists. Among mammalian GALT species, the rabbit is the best studied (Lanning and Knight 2015). In the rabbit *IgH* gene, although more than 200 VH genes are present, in most cases only the V gene closest to the *D* segments undergoes rearrangement. The nucleotide sequence of the *VDJ* unit is further modified by gene conversion; however, unlike chickens, in rabbits, SHM also contributes significantly to the diversification of the primary repertoire. Interestingly, when the appendix, the rabbit GALT tissue specialized in the formation of the primary B repertoire, is deprived of microbiota, a significant depletion in the primary Ig repertoire is observed (Lanning and Knight 2015). Intense research is underway on molecules from the microbiota, likely super‐antigens, which somehow promote the formation of functional B lymphocytes in the rabbit. Interestingly, the antibody diversification strategy used in GALT species is conserved in a subset of human and mouse B cells (Lanning and Knight 2015).

In summary, the Ig rearrangement dynamics found in the human and mouse species are present only in rodents and primates (so‐called PR group) (Flajnik 2002), and most mammals follow the pattern of GALT species. Therefore, by a fortunate chance, the mouse and rat constitute optimal models for studying the human immune response.

In cartilaginous fish, such as sharks, rays and skates, *Ig* loci are organized in clusters (miniloci). In the different species studied, the number of clusters varies widely. Three IgH isotypes are found in these animals: IgM, present in most vertebrates; IgW, orthologous to mammalian IgD; and IgNAR, an Ig class characteristic of this group of animals, which forms antibody molecules consisting only of heavy chains (Matz et al. 2020). Although the configuration of *Ig* loci and the organization of lymphoid tissue in cartilaginous fish are very different from those of other vertebrates, the B responses turn out to be remarkably similar functionally. That is, B lymphocytes are monoclonal and secrete only one Ig isotype at a time. Moreover, antibody responses in these animals are characterized by great diversity, affinity maturation and memory (Matz et al. 2020). Cartilaginous fish use similar recombination mechanisms as mammals to generate Ig diversity, and rearrangement is mostly restricted within clusters. In analogy to what occurs in mammals, the primary repertoire derives from combinatorial (though in these basal vertebrates, it is the number of miniloci that matters) and junctional diversity. AID‐driven SHM plays a role only during the establishment of the secondary Ag‐dependent repertoire (Matz et al. 2020).

Although the apparatus for forming antibody responses may differ across vertebrate classes, the functional outcomes are strikingly similar. The reasons for this functional convergence are the subject of intense study. Differences found among the various species are partly the consequence of taxonomic position and evolutionary divergence and partly due to ecological constraints. Indeed, species belonging to distant taxonomic groups may display striking similarities. This is, for example, the case in cartilaginous fish and camelids, which, in parallel with conventional tetrameric Ig, also produce dimeric Ig consisting only of heavy chains. As Ig formed from heavy chains alone are more stable and resistant to denaturation, it has been hypothesized that this evolutionary convergence is due to the shared extreme conditions in the blood stream, such as high urea concentrations in both cartilaginous fish and camelids, and high blood temperature in camelids (Flajnik et al. 2011).

## 3 TCR: An Antigen Receptor More Conservative Than BCR/Ig Due To MHC Restriction

Comparative studies across various vertebrate classes have contributed significantly to highlighting the differences between Ig/BCR and TCR (Flajnik 2018). B responses demonstrate remarkable developmental and functional plasticity, in contrast to T responses, which appear more conservative. It allows for the construction of a coherent and simplified model encompassing many biological aspects, from the organization of the loci encoding the two receptors, to the anatomy of the organs involved and their function.

1. In cartilaginous fish, *Ig* loci are organized in clusters (multiple miniloci). In bony fish, there is a mixed organization, with translocon (*IgH*) or cluster (*IgL*) arrangements, whereas in all tetrapods, the organization is exclusively in translocons. In contrast, TCR loci are consistently organized in translocons from cartilaginous fish to mammals (Flajnik 2018).
2. Although the divergence of major Ig isotypes is ancient, with IgM present in all gnathostomes, various vertebrate classes have evolved unique, clade‐specific antibodies that do not resemble IgM, IgD/W, IgY/IgG/IgE or IgA. In contrast, in all vertebrate classes starting with cartilaginous fish, only four TCR chains—TCR‐α, TCR‐β, TCR‐γ and TCR‐δ—are consistently found (Flajnik 2018).
3. The recently discovered exception includes monotreme and marsupial mammals, where, alongside the canonical TCR isotypes, there is a TCRμ isotype (Wang et al. 2011).
4. The primary lymphoid organ responsible for B lymphocyte maturation varies across vertebrate phylogeny and during ontogeny. In contrast, T lymphocyte maturation consistently occurs in the thymus, which is present across species from cartilaginous fish to mammals (Boehm et al. 2013).
5. For Ig, there is a primary repertoire generated by recombination at the *V(D)J* junction or alternatively by SHM and/or gene conversion. This repertoire is further diversified and refined in secondary lymphoid organs through SHM and/or gene conversion. In contrast, TCR diversity arises exclusively during the ontogenetic maturation phase, through *V(D)J* recombination. Once T cells exit the thymus, no further changes occur in TCR loci, effectively locking the TCR repertoire. It has been stated that B lymphocytes ‘learn life from the street’ (Townsend et al. 1999). T cells, by comparison, have a ‘Victorian upbringing’. B lymphocytes recognize antigens in their free form, whereas T lymphocytes recognize antigens that are presented in association with highly polymorphic self molecules (MHC). This makes the T cell system more conservative due to MHC restriction and the co‐evolution that has shaped it. Self‐reactivity is also more functionally significant in the T cell compartment, necessitating a stricter ‘education’ process compared to B cells (Janeway 2001).

This simple and coherent view has recently been partially challenged (Ott et al. 2021). Cartilaginous fish, the most basal group of extant gnathostomes, exhibit a higher degree of plasticity in T cell biology than previously thought. These vertebrates diversify their TCR repertoires through the formation of non‐canonical TCRs that incorporate gene elements of both TCR and BCR (*IgH*‐TCR trans‐rearrangements). Additionally, TCR genes in cartilaginous fish undergo AID‐dependent SHM. However, consistent with the classical understanding, SHM in TCRs occurs exclusively in the thymus, likely serving to further modify primary rearrangement products for successful thymic selection, functioning as a receptor editing mechanism (see Box S3). Research on cartilaginous fish has been pivotal, as similar TCR plasticity has since been observed in various other vertebrate taxa.

## 4 Alternative Mechanisms for High Diversity Immune Receptors: Lessons From the GOD Debate

Following the discovery, around the late 20th century, of AID and the elucidation of mechanisms underlying SHM and isotype switching, the characterization of the diversity generation in Ig/BCR and TCR receptors in model mammals has reached a comprehensive level. However, research into vertebrates that are evolutionarily more distant has unveiled novel and sometimes surprising mechanisms of diversity in Ig/BCR and TCR. Even more strikingly, in the last 25 years, immune defence systems with functional similarities to acquired immunity have been identified in taxa outside of jawed vertebrates. This finding challenges the long‐held belief that acquired immunity was exclusive to gnathostome vertebrates (Jack and Du Pasquier 2019).

One notable example is the discovery of VLRs in jawless vertebrates by Max Cooper (Herrin and Cooper 2010). Beyond jawless vertebrates, various genes involved in generating extensive receptor diversity have been characterized in different invertebrate phyla (Li 2021; Adema 2015; Ghosh et al. 2010). Therefore, the idea has been gaining support that the acquired immunity of jawed vertebrates, known as RAG‐dependent immunity, represents just one possible form present in the tree of life. Our claim is that the debate sparked by the antibody diversity paradox in the first 75 years of the 20th century generated several foundational concepts that remain relevant today, providing guidance for the characterization of newly discovered anticipatory immune systems.

1. A classic argument, anticipated by both opponents of Ehrlich's side chain theory (Silverstein 2003b) and advocates of somaticist theories (Silverstein 2003a), is about the selective pressure that maintains in the genome large numbers of gene segments, many seldom used, specific for particular pathogen‐associated molecules.

This question was revisited in the 1970s and analysed in detail through the lens of the evolutionary dynamics of multigene families. Theoretical considerations suggest that homogenizing forces typically operate within multigene families, and a classic example of this concerted evolution is seen in the hundreds to thousands of ribosomal DNA (*rDNA*) gene units (Wang et al. 2023). Conversely, there is no indication of concerted evolution in Ig coding genes, where strong diversifying selection preserves a broad germline *V* gene segment repertoire (Ota et al. 2000).
Comparative genomics analysis indicates that the variability in Ig *V* gene fragments is organized into families and subfamilies, which have remained stable over several 100 million years (Andersson and Matsunaga 1995). Moreover, it has been shown that knocking out single *V* gene fragments in Ig genes makes mice more vulnerable to specific bacterial infections. This suggests that selection pressure maintains a large repertoire of pathogen‐specific, functionally relevant *V* fragments which are associated with key neutralizing epitopes (Mi et al. 2000). At present, it is entirely plausible that the genome can maintain a large repertoire of receptors, which likely originated from the evolutionary arms race between the host's ancestors and their parasites. This idea, which is widely supported in vertebrate immunity and aligns with the germinalist perspective, could potentially be applied to any gene system involved in newly characterized forms of anticipatory immunity.

1. In arthropods, a gene has been characterized that can generate a wide diversity of specific receptors for pathogens or pathogen‐associated molecules (Brites and Du Pasquier 2015; Li 2021).

The Down Syndrome Cell Adhesion Molecule (*Dscam*) gene produces over 10,000 different isoforms through alternative splicing. These Dscam products are expressed either as membrane‐bound molecules or, alternatively, as soluble molecules. At the single‐cell level, Dscam expression is most likely a fraction of total diversity generated by alternative splicing; namely, it is oligoclonal. Functional studies have shown that Dscam mediates pathogen‐specific phagocytosis and contributes significantly to immune defence responses. Furthermore, Dscam appears to exhibit a form of immune memory. It has been suggested that pathogen‐specific, membrane‐bound Dscam may transduce a signal to induce the expression of soluble Dscam at the infection site. However, the mechanism by which the pathogen selects or induces pathogen‐specific alternative splicing remains largely unclear.
The current understanding, though somewhat inconsistent and requiring further clarification, suggests certain similarities between arthropod Dscams and vertebrate antibodies.
It is important to highlight a few key points: *Dscam* diversity is entirely germline‐encoded and orders of magnitude lower than the diversity seen in antibodies; Dscam expression is oligoclonal, not monoclonal; haemocytes in arthropods do not proliferate after microbial challenge, so no clonal expansion occurs. Of note, these points echo Ehrlich's model of antibody generation.

1. Somewhat unexpectedly, the instructive theories proposed in the first half of the 20th century find resonance in modern immunology. In this case, it is not the 3D structure of an antigen but the nucleic acid sequence of a viral pathogen that serves as a template for immune adaptation. This concept is exemplified by mechanisms such as CRISPR‐Cas in bacteria and archaea (Nussenzweig and Marraffini 2020) or with RNA interference in various organisms, both animals (Schuster et al. 2019) and plants (Akbar et al. 2022).

Indeed, in these defence systems, the host genome is modified through the insertion of foreign DNA derived from viral pathogens. Following transcription, these foreign sequences then target and destroy the genome of invading viruses. These forms of immune protection are highly specific, acquired and, in some cases, heritable across generations. As such, they are often referred to as Lamarckian immunity due to their potential for inheritance and adaptive changes across generations (Flegel 2009; Koonin and Wolf 2016; Müller et al. 2018).

## 5 Concluding Remarks

Antibody diversity has been one of the most captivating topics in the life sciences for over 70 years. This article has primarily focused on the theoretical debate surrounding the antibody enigma, a topic that has played a pivotal role in driving experimental investigations. In the early stages, from the early 20th century to the 1950s, the theoretical debate was marked by the opposition between selectionist and instructionist theories. Later, this contention evolved into a debate between germinalists and somaticists. After 1957, the instructionist positions were largely dismissed.

However, a major point of this article is to emphasize that in scientific inquiry, opposing positions are rarely entirely wrong or sterile. The resolution of most scientific debates often involves partial validation of the basic assumptions of both sides. As Silverstein has noted in the context of immunology research, this pattern of integrating seemingly contradictory viewpoints is a hallmark of scientific progress (Silverstein 2009).

In this article, we have discussed the mechanisms underlying Ig and TCR gene diversity, or the ‘nature’ of GOD. However, we have not addressed the other related topic of the ‘origin’ of GOD. This area of investigation has sparked a theoretical debate that largely runs parallel to the one discussed here, focusing on the domestication of transposon elements coding for the RAG recombinase (Zhang et al. 2019) and the ‘big bang’ emergence of RAG‐dependent acquired response (Schluter et al. 1999; Flajnik 2014; Müller et al. 2018). This idea, although somewhat controversial due to its divergence from gradualism—a mainstay of Darwinian evolution—(Klein and Nikolaidis 2005, Dzik 2010)—remains important in discussions of immune system evolution.

In conclusion, comparative biology studies show that species derived from different evolutionary histories but exposed to similar environmental pressures can sometimes achieve substantially similar functional outcomes, even though mechanisms are very different. These evolutionary convergences, which are seen in numerous anatomic‐functional systems (Blount et al. 2018), are also found in the immune system.

## Conflicts of Interest

The authors declare no conflicts of interest.

## Supporting information



Supporting Information

## Data Availability

The data that support the findings of this study are available from the corresponding author upon reasonable request.
