# Supplementary material for: The Quest for Antibodies and Other Acquired Immune Receptors: A Historical Perspective
Source: Int J Immunogenet. 2025 May 8;52(3):125–34. doi: 10.1111/iji.12712 (PMC12087747; doi:10.1111/iji.12712)
Supplement: Supplementary file 1 — Supporting Information [file IJI-52-125-s001.docx]

**Box1**

**BOX1, TIMELINE *antibody and TCR research placed in the historical context of immunological research***

**historical context**

with some level of approximation, it is possible to divide immunological research into three periods:

-**from 1890 to 1957,** **“dark ages of immunology”** the beginning of immunology as a discipline is placed in 1908, the year the Nobel Prize was awarded to Metchnikov and Ehrlich (**Kaufmann 2008**). but the first evidence for the existence of antibodies dates back more than 15 years earlier. The designation “dark ages of immunology”, to indicate the first phases of research in immunology, was introduced in Zoltan Nagy's book (**Nagy 2013**). Research was dominated by serology, immunology was predominantly a translational discipline, the immunochemical approach being the most successful. Functional evidence suggested biological complexity that was unsatisfactorily explained by the models in vogue at the time.

-**from 1957 to 1989**, **modern immunology** In 1957 Burnet advanced the theory of clonal selection, widely regarded as a turning point. From this time onwards, biological/functional aspects were central to the discipline, with massive reliance on cell biology and genetics and nascent molecular biology. Crucial was the availability of the inbred mouse strains developed in histocompatibility studies in the 1930s (**Mc Devitt 2000**). The main questions that aroused the curiosity of researchers pertained to acquired immunity and were the specificity and diversity of B and T responses, tolerance to self and its failure in autoimmune diseases.

-**from 1989 to the present day, contemporany immunology** 1989 was the year Janeway published the theory of pattern recognition (**Janeway 1989**), regarded as a second turning point. The interest of the scientific community is mostly focused on innate immunity (**Vivier and Malissen 2005**). The experimental toolbox was enriched by increasingly sophisticated techniques in *in vivo* and *ex vivo* cell biology and molecular biology; later, omics, supported by bioinformatics, revolutionized the field, enabling greater interdisciplinarity and holistic approaches. A wider range of questions and sub-disciplines become the subject of intense research.

**research on acquired immunity receptors**

**1890-1976** In the first half of the 20th century the paradoxical attributes of antibody responses gradually emerged; 1957, the year of publication of the theory of clonal selection, closed the debate between instructive and selectionist theories in favor of the latter. The 1960s were times of intense experimentation but also of lively theoretical debate. During this period, awareness for the enigma of antibody diversity grew enormously in the scientific community. In 1976 Tonegawa produced the first evidence for the process of somatic recombination of antibody-coding genes in the B cell lineage (**Tonegawa 1988**). The timeline in the period 1890-1976 is mostly intended to place chronologically the major theoretical contributions to GOD debate.

**1976-2020s** Since 1976, research has entered a new phase, the progressive application of molecular biology techniques enabled the determination of the fine structure (sequence) of genes encoding antibodies within a few years(**Tonegawa 1988**). In the early 1980s, the TCR, whose existence had been hypothesized many years earlier, was finally identified (**Reinhertz 2014**), and to follow, the molecular machinery driving somatic rearrangement (RAG1/RAG2, TdT) (**Brandt and Roth 2008**), somatic hypermutation (AID)(**Honjo 2008**), and isotype switching (AID) (**Honjo 2008**), was elucidated. The study of the mechanisms underlying BCR/Ig and TCR diversity did not end with model mammals, mice and humans, but continued in vertebrates farther and farther down the evolutionary chain (**Jack and Du Pasquier 2019**). This vast research program, part of comparative immunology, has no clear temporal boundaries having started as early as mid 1960s and continuing to the present day. Of note pioneers in early characterization of Ig molecules in lower vertebrates and comparative immunology received their training in the laboratories of leading immunologists; Marchalonis was a student of Edelman and Litman was a student of Robert Good. Another renowned protégé of Good was Max Cooper, who first characterized B lymphocytes as directly involved in antibody production through his famous bursectomy experiments in the chicken (**Cooper 2010**). At a late stage in his career, Cooper was the protagonist of the discovery of a parallel system of acquired immunity in agnate vertebrates, in many ways functionally equivalent to acquired immunity in gnathostome vertebrates but based on VLRs receptors, consisting of LRR modules instead of Ig-like modules characteristic of antibodies, TCRs and MHC molecules (**Cooper 2010**). Further characterization of antibody-coding genes and even of TCR, at the protein and gene structure level, came in the late 1970s when the necessary molecular biology tools - immunoscreening of expression libreries using species specific antibodies, DNA-cross-hybridization using probes from higher vertebrates, PCR using mixed primers based on short conserved regions in the Ab genes - were developed (**Bilal et al 2021**). After these laborious early efforts, the rapid development of DNA sequencing methods and advances in bioinformatics have enabled the study of acquired immunity receptors in a broad variety of vertebrate species relevant for their place at branch points of phylogeny or for their ecology. Leading figures in comparative immunology of vertebrates in the last 30 years are Louis Du Pasquier and Martin Flajnik while in the most recent generation of researchers in this field, Katherine L Knight can be mentioned. At last, the scientific community began to investigate the rise of acquired immunity receptors (origin of GOD) by extending the analysis to basic chordata and invertebrate deutorostomes (**Zhang et al 2019**). These studies, also extended to invertebrate protostomes, demonstrating the existence of alternative forms of adaptive/anticipatory/acquired immunity (**Müller et al 2018**).

**Box2**

**Contribution of Paul Ehrlich and Frank Macfarlane Burnet to the theoretical debate on antibody diversity**

**EHRLICH**

Paul Ehrlich (1854–1915) was a German physician whose training in the laboratory of the famous organic chemist and enzymologist Emil Fischer was highly significant. Fischer, who first introduced the metaphor of the key-and-lock interaction to explain enzyme catalysis, influenced Ehrlich’s lifelong interest in the relationship between chemical structure and biological function. Ehrlich made foundational contributions to the nascent field of immunology and developed early forms of chemotherapy for bacterial infections. In recognition of his achievements, he shared the 1908 Nobel Prize in Physiology or Medicine with Elie Metchnikoff—a milestone some consider marking the birth of modern immunology (**Kaufmann 2008**).

Ehrlich introduced the term antibody in the German language, which later became widely used in English and replaced the previously used term anti-toxin (**Lindenmann 1984**). His side-chain hypothesis is the first selectionist theory to explain antibody diversity. Ehrlich postulates the existence of a large but finite number of side chains that specifically recognize “nutrients” and may also cross-react with bacterial and environmental toxins. According to the theory, there could also be pre-formed side chains directly specific to toxins. Each cell simultaneously produces side chains of various specificities; moreover, Ehrlich did not assume the existence of cells specialized in the exposure of side chains. He proposed that the abundant secretion of antibodies following an antigenic challenge was an example of over-compensation, a concept introduced by his cousin, the pathologist Carl Weigert, to explain several phenomena observed in pathology.

Initially, the theory enjoyed some favor within the scientific community, as it did not face the problem later posed by the existence of an overly large repertoire of antibodies. However, the inconsistencies of the theory soon emerged. Max von Gruber, a professor of hygiene in Vienna and mentor to Karl Landsteiner, harshly criticized Ehrlich’s proposal. Gruber questioned how evolution could have led to the development of so many side chains specific to substances that an organism might never encounter. What selective force would act to preserve such unlikely specificities (**Gruber 1903**)?

The debate between Ehrlich and Gruber soon degenerated into acrimonious polemics (**Silverstein 2009**), Gruber must be credited with being the first to raise an issue that would become the subject of considerable debate. So ironically, Darwinism was invoked at the time as an argument against, rather than in favor of, the side-chain theory. In 1914, a year before Ehrlich's passing, the physician and bacteriologist Hans Zinsser concluded in his survey of the topic, that the theory of side chains was outdated and, at best, held only, historical interest (**Zinsser 1914**).

It is crucial to note that in the medical field and, more broadly, in functional biology, Darwinian and Lamarckian positions were often adopted somewhat unconsciously during the first half of the 20th century. This was the case for Ehrlich, whose thinking was fundamentally Darwinian, as well as for Breitl Haurowitz and Linus Pauling, who espoused a loosely Lamarckian view (**Silverstein 2003b**).

On the other hand, Metchnikoff explicitly and consciously referred to Darwinism. However, it is important to note that the Russian scholar was trained as an embryologist and a zoologist, not as a physician or a chemist. Moreover, Metchnikoff’s task was relatively simpler, as he assumed an evolutionary dynamic - an “arms race”- between organisms of different species: parasites and their hosts. In contrast, the evolutionary dynamics needed to explain the paradox of antibody diversity occur within the organism, between molecules and/or cells. It is reasonable to say that the time was not yet ripe for this conceptual leap.

With the benefit of hindsight, Ehrlich’s theory of clonal selection anticipated several fundamental concepts in modern biology:

a)Along with the neurophysiologist John Newport Langley (**Prüll 2003**), he was first to hypothesize receptor-ligand interactions, a concept of outstanding impact in biomedicine, in pharmacology, and the life sciences.

b) He suggested a cell-associated receptor that, upon cell activation, is over-produced and released into the extracellular milieu as a soluble receptor.

c) He introduced the investigation of Darwinian dynamics within the organism, specifically within populations of cells exhibiting variable genotypes and phenotypes. This concept is now applied in the acquired immune response, cancer research (**Nowell 1976**) and even in neural network wiring

(**Edelman 1987**) although the latter application of Darwinian dynamics at the cellular level within the organism remains controversial(**Crick 1989**).

d) He was instrumental in introducing terms and metaphors later widely adopted by the scientific community, such as “antibody”, “receptor”, “magic bullet” and others.

**BURNET**

The Australian Frank Macfarlane Burnet (1899-1985) initially gained recognition for his outstanding contributions to virology.

In the early 1940s, Burnet’ first approach to immunology was largely theoretical, his challenging goal was to develop a coherent view of how the immune system works. As a result, Burnet addressed the antibody diversity paradox and introduced the concepts of immunological tolerance and self-nonself-discrimination. For his contribution to the understanding of immunological tolerance, Burnet was awarded the Nobel Prize in 1960, along with the British biologist Peter Medawar, who provided experimental support for the theory.

At first, being more acutely aware than most contemporary immunologists of the limitations and inconsistencies of the instructive theories, Burnet attempted to modify these hypotheses to make them more consistent with biological/functional evidence. In the first edition of his monograph on the subject (**Burnet et al 1941**), Burnet employed the concept of the *adaptive* enzyme, borrowed from bacterial genetics (**Dubos 1940**). The antigen that enters the body is channeled into the reticuloendothelial system, where it induces the synthesis of an enzyme -from time to time different- which in turn assembles the Ag-specific, cognate antibody. All this occurs in a cell that proliferates while maintaining the capacity to produce and secrete antibodies. Therefore, the specific antibody continues to be synthesized even after the antigen is no longer present. Moreover, this model helps explain the logarithmic increase of antibodies during the secondary response. This proposal was an *indirect template theory* because the antigen impresses a complementary pattern; in other words, it acts as a template, not *directly* on the globulin molecule, but *indirectly* on a cellular process. A few years later, in 1949, Burnet published a second edition of the monograph with the virologist Frank John Fenner (**Burnet and Fenner 1949**) in which he revised his hypothesis. According to the new version of the indirect template theory, the antigen induces a change in the store of information located within the cell, which today we would refer to as the genome. Indeed, within a few years, the idea that enzymes could be adaptively modified had lost credibility while increasing importance was placed on nucleic acids, particularly RNA, which Jean Brachet had shown to be involved in protein synthesis (**Brachet and Chantrenne 1956**). It is important to emphasize that the speculations formulated by Burnet in the 1940s are fundamentally Lamarckian, as they imply a form of inheritance of acquired characteristics at the cellular level. The great credit given today to the Danish immunologist Niels Kay Jerne is that he has revisited Ehrlich's ideas and formulated a selectionist, explicitly Darwinian hypothesis to explain antibody diversity **(Jerne 1955)**. In a sense Jerne broke the deadlock. In brief, Jerne’s natural selection theory posits that when the antigen enters the organism, pre-existing antibodies interact with it and the Ag/Ab complex is captured by cells of the immune system, where the antibody is then reproduced. The difference between Burnet's template hypothesis and Jerne's new hypothesis lies in in the first stage of the process: instructional in the former case and selectionist in the latter. The second step of the process – namely, the production within the cell of the specific antibody - is essentially similar in both theories; they both imply a flow of information from the outside to the genetic apparatus of the cell, which aligns them more with a Lamarckian view.

Two years later, in 1957, Burnet published a paper that explicitly, modified Jerne's theory, as reflected in the title (**Burnet 1957**). Jerne speculated that the first encounter between Ab and Ag occurs in the fluid phase, after which the complex is transported to a cell that overproduces the specific antibody. In contrast, Burnet hypothesized that the first encounter between Ab and Ag occurs on the surface of the cell, which, upon activation, overproduces the Ab. Maybe the major strength of Burnet's proposal lies in its potential consistency with the emerging vision of protein synthesis, particularly the work of molecular biologist Francis Crick (**Crick 1958**). Burnet also acknowledged the contribution of the American immunologist Davis Wilson Talmage, who had published a review on allergic diseases (**Talmage 1957**) that anticipated some elements of the clonal selection theory, but did not elaborate on them. Burnet advanced this idea significantly by explicitly referencing population biology, introducing the term "clonal selection," and, most importantly, hypothesizing that the antibody-producing cell exposes an immunoglobulin specific for a single antigen on its surface.

The implications of the clonal selection theory were examined more fully in a series of lectures presented at the Vanderbilt University in Tennessee, which were later published, in 1958, in a groundbreaking monograph by Cambridge University Press (**Burnet 1959**). After 1957, Burnet reorganized the experimental research projects in the institute, shifting from virology to functional immunology. Burnet and his collaborators, most notably Gustav Nossal, designed experiments to test and/or falsify the theory and sought to explore its power, particularly in explaining the ontogeny of immune responses. Moreover, throughout the 1960s, Burnet took part in the debate between somaticists and germinalists, advocating for the former. He argued, along with the American molecular biologist Joshua Lederberg, that antibody diversity is primarily due to a high mutation rate in Ig coding genes.

In summary, several areas of research contributed to the development of the now substantially confirmed theory of clonal selection: functional immunology, genetics of microorganisms, and nascent molecular biology. The theory adopted the concept of clone from the genetics of microorganisms. Moreover, the Luria-Delbrück experiment of 1943 was also highly influential in shaping the theory (**Luria and Delbrück 1943**). It was shown in the Luria-Delbrück experiment that the "bacteriophage T1 resistance" character in E. coli preexists in very few bacteria even before exposure to phage T1. Luria and Delbruck's paper is remembered as one of the first experimental evidence of Darwinism. In support of the close connection between the two research areas, Jerne’s paper was presented on the Proceedings of the National Academy of Sciences (PNAS) by Delbruck himself, two years earlier.

The other major prerequisite and constraint for the theory was the mechanism of protein synthesis. In fact, any theory of antibody formation had to account for the mechanism of protein synthesis. In 1958, Crick published a landmark article (**Crick 1958**) in which a unidirectional flow of biological information from DNA to RNA to protein is hypothesized. In addition, Crick, in further support of his proposal, later called the central dogma of molecular biology, cited the work of the biochemist Christian Anfinsen (**Anfinsen et al 1961**), who had shown that the folding, and thus the catalytic activity, of RNAse depends entirely on the amino acid sequence and, ultimately, on the structure of the gene encoding the enzyme.

In contrast to earlier theories, Burnet's 1957/1958 proposal is compatible with the central dogma. By interacting with the cognate antibody in the membrane, the antigen determines, through signal transduction mechanisms - intensively investigated later - the over-expression of the corresponding coding gene already present in the cell nucleus.

A final aspect of the formulation of the theory, perhaps less crucial but still important, is the nature of the cell that produces and secretes antibodies. By the 1950s, the scientific community had accepted that such a cell was the plasma cell, identified in 1947 by the Swedish immunologist Astrid Fagraeus (**Fagraeus 1947**). However, the origin of the plasma cell was controversial. Several immunologists believed that the plasma cell was derived from a macrophage-like cell within the reticuloendothelial system consistent with the idea that antigen is internalized by a cell where it induces antibody synthesis, a concept in line with template theories. Other data, however, indicated the involvement of small lymphocytes in antibody responses suggesting that plasma cells are derived from lymphocytes. This latter view prevailed later.

In conclusion, the theory of clonal selection suggested the existence of an unprecedented randomization mechanism operating at the level of genes encoding antibodies. Research developments over the next fifthy years have essentially confirmed Burnet's hypothesis by highlighting two molecular processes a)gene rearrangement, located in the primary lymphoid organs and driven by RAG1/RAG2 recombinases, resulting in the primary repertoire b)somatic hypermutation, located in the secondary lymphoid organs and catalysed by activation-induced deaminase AID, yielding the secondary repertoire (**Neuberger 2008**).

**Box3**

**legend to figure**  **A)** *IgH* gene in germline configuration, in rearranged configuration and after somatic hypermutation and Cµ/Cα class switch recombination **B)** magnification of *VDJ* rearranged exon/module in which the position of CDR1, CDR2 and CDR3 is highlighted. CDR1,CDR2 and CDR3 form bulging loops in the IgV globular domain; CDR1, CDR2 and CDR3 in IgH and IgL form the paratope of antigen binding (not shown)

**Diversity and plasticity of Ig and TCR responses in model mammals**

***Germline organization and rearrangment, formation of the Ig primary repertoire***

In humans and mice, three Ig loci exist. A single gene for the heavy chain IgH, which encodes for all immunoglobulin isotypes, and two distinct loci encoding the light L chain, one encoding the κ chain, IgLκ, and the other encoding the λ chain IgLλ.

The gene organization of the *IgH* is an examples of the so-called *traslocon configuration*, from 5' to 3': an array of *V* gene fragments, V1 to Vn; an array of *D* gene fragments, D1 to Dm; an array of *J* gene fragments, J1 to Jr and finally the gene segments encoding the C domains.

During B-lymphocyte ontogeny, a process of nonhomologous recombination takes place involving in temporal order first the *IgH* locus and then either of the two *IgL* loci. The D-J rearrangement “welds” a randomly taken D fragment with a randomly taken J fragment, follows the V-DJ rearrangement between a *V* gene fragment and the *DJ* unit produced in the previous step. At this point the gene can be transcribed, the primary transcript goes through splicing, and finally the mature messenger is exported from the nucleus to the cytoplasm and translated into the heavy chain polypeptide. Somatic recombination results in the formation of circular DNA molecules, the excised DNA, basically waste products; accordingly *Ig* coding genes get shorter.

Of note the junction point between the *V* and *J* gene segments is not “sharp” but is subject to insertion or removal of a small number of nucleotides. This fact gives rise to a significant number of unproductive rearrangements because out-of-frame, but is not an accident along the way rather an integral part of the rearrangement process; in fact it greatly increases the diversity of receptors of acquired immunity.

The general dynamics of the rearrangement process suggests answers to the long-lasting enigma of antibody diversity. The multiplicity of *V*, *D* and *J* fragments, and subsequently the random assembly of light and heavy chains, produces a *combinatorial diversity* of about 2.5 x10^6^. In reality, the estimated primary Ig repertoire is orders of magnitude higher due to the *junctional variability* at the joining points between *V D J* gene fragments.

In both light and heavy chains, there are three regions of hypervariability in the V domain, called Complementary Determining Region CDR1,CDR2 and CDR3.

The NH2-terminal variable domain of Ig results largely encoded by the *V* segment, about 300 nucleotides long (the first 100 aa), while the *D* and *J* gene fragments, for the heavy chain, or *J* for the light chain, contribute the last approximately 10 COOH-terminal amino acids. CDR3 is located across the joining site involved in rearrangement instead CDR1 and CDR2 are within the *V* gene segment. The contact surface between the antibody and the antigen consists of *at most* six CDRs, forming bulging loops, three from the heavy chain and three from the light chain. The molecular portion of the antigen directly recognized by the antibody is termed the *epitope*, whereas the antigen-binding site on the antibody is termed the *paratope*.

In summary, combinatorial diversity is greater the more numerous the number of *V*, *D*, *J* fragments in the genome; junctional diversity is expressed at the level of CDR3, which varies by sequence but also by length. It should be emphasized that combinatorial diversity supports the germinalist view instead junctional diversity supports the somaticist view, this is because the nucleotides inserted in the V-D D-J or D-J junctions are not encoded anywhere, they are nucleotides put there randomly.

The somatic recombination process is driven by short conserved sequences flanking the *V, D, J* fragments called *Recombination Signal Sequence* (RSS). Numerous proteins operate at the level of these cis elements, among them most belong to ubiquitously expressed enzymes of the nonhomologous end-joining (NHEJ) pathway (or are borrowed from meiotic recombination), some, however, are expressed exclusively at the ontogenetic stages in B cell lineage when recombinations occurs. So they are highly specific and serve as the molecular signature of the recombination process. These are *Recombination-Activating* *Genes RAG1/RAG2*, with endonuclease activity that “open” the DNA at RRS points and *Terminal deoxynucleotydyl Transferase (TdT)* that randomly adds nucleotides to the ends of single-stranded DNA.

At Ig loci, transcription is under the influence of enhancer regions located upstream and/or downstream of the group of exons encoding the constant portion of the molecule. As a result, Ig transcription is active exclusively on the V(D)J module directly involved in rearrangement because the promoter region “gets close” to the enhancers. Monoclonality of B lymphocytes is due to an allelic exclusion mechanism whereby rearrangement at the Ig loci present on one chromosome somehow inhibits rearrangement on the other chromosome. In addition, receptors originating from the rearrangement can be expressed on the membrane as B cell receptor (BCR) or alternatively be released as soluble Ig through an alterative spicing mechanism.

The anatomical site of somatic rearrangement is the primary lymphoid organ, the bone marrow in the adult, and the cells in which this process takes place are the immature B-lymphoid precursors. This process is entirely independent of the interaction of the B precursors with antigenic material of any nature or origin, self or non-self. The rearrangement generates the so-called *primary* or naive B repertoire.

As a main functional requirement, the developing B cells undergo negative selection that eliminates cells that recognize self epitopes, consequently mature B cells that reach the bloodstream in most cases are tolerant to self. A process of *receptors editing* increases the likelihood to get functional B cells by rescuing those lymphocytes that have carried out unsuccessful rearrangment or that produce an autoreactive Ig. After multiple failed attempts of receptor editing, the cell eventually goes into apoptosis.

***Somatic hypermutation and affinity maturation, formation of secondary Ig repertoire followed by class switch recombination***

From the bone marrow, mature but naive B lymphocytes reach the secondary lymphoid organs -lymph nodes, spleen, GALT- through the blood circulation. In those anatomical sites further antibody diversity is generated in the naive B lymphocytes that have encountered for the first time the antigen, in other word that have gone through *priming*. Accodingly, rearranged *Ig* genes undergo a diversification process called somatic hypermutation which consists of a high frequency of point mutations scattered within the V(D)J rearranged segment. Somatic hypermutation is a largely random process, but is somewhat driven by the interaction with the antigen. In this case, mutation and selection are coupled: the antigen present on the cell surface of follicular dendritic cells, placed in germinal centers of secondary lymphoid organs, selects for variants of the original antibody generated by hypermutation endowed with increased affinity. This process leads to affinity maturation in the B cell response. In this way somatic hypermutation generates the secondary B cell repertoire.

In addition to affinity maturation, in secondary lymphoid organs, immunoglobulin class switch from initially expressed IgM to IgG or IgA or IgE also takes place aiding functional plasticity to the Ig wide repertoire. The isotypic switch consists of a change in the overall structure of the gene encoding the immunoglobulin heavy chain, due to the deletion of long gene tracts; the IgH gene is shortened further.

The factors and enzymes that drive somatic hypermutation and Ig class switch are partly the same. In both these processes the key, signature enzyme is *Activation Induced Deaminase AID* assisted by accessory factors; AID is a DNA mutator enzyme belonging to the *AID/APOBEC* family.

In conclusion, the primary Ig repertoire is RAG-dependent whereas the secondary Ig repertoire is AID-dependent.

***-TCR and T Cell biology***

In Vertebrates there are two types of acquired immunity receptors, BCR/Ig expressed on B lymphocytes and TCR expressed on T lymphocytes. Both of these receptors consist of tandem repeats of protein domains belonging to the Ig super-family. Also in the case of TCR, somatic rearrangement follows essentially the same patterns and is based on the same cis- and trans-elements described above for B lymphocytes, generating a very large repertoire of antigen receptors.

T lymphocytes constitute a broad category of cells belonging to acquired immunity or operating at the interface between innate and acquired immunity; two subsets of T lymphocytes exist, expressing TCRαβ or TCRγδ, respectively; the theory of clonal selection also applies in the case of T lymphocytes, TCR expression is monoclonal due to an allelic exclusion mechanism.

There are substantial differences between BCR/Ig and TCR: a)TCRs are expressed exclusively as membrane receptors. b) The diversity of TCRs is only due to gene rearrangement, thus to combinatorial and junctional diversity; indeed, in the case of TCR, there is no SHM, affinity maturation, and thus no secondary repertoire. c) TCRαβ T lymphocytes recognize exclusively protein ligands, never in native or denatured form but processed into peptides. Antigenic peptides are presented to TCRs in association with MHC-I and II molecules characterized by broad allelic polymorphism (MHC restriction). In contrast, TCRγδ bind ligands of various chemical nature, often relatively invariant in analogy with receptors of innate immunity; moreover T TCRγδ cells often bind free-phase antigens in a manner similar to B lymphocytes or antigens associated (presented by) with monomorphyc MHC-like molecules.

Anfinsen, C. B., E. Haber,M. Sela, and F. H. White Jr. 1961. “The Kinetics of Formation of Native Ribonuclease During Oxidation of the Reduced Polypeptide Chain.” PNAS 47: 1309–1314.

Bilal, S., A. Etayo, and I. Hordvik. 2021. “Immunoglobulins in Teleosts.”Immunogenetics 73, no. 1: 65–77. <https://doi.org/10.1007/s00251-020-01195-1>.

Brachet, J., and H. Chantrenne. 1956. “The Function of the Nucleus in the Synthesis of Cytoplasmic Proteins.” Cold Spring Harbor Symposia on Quantitative Biology 21: 329–337. <https://doi.org/10.1101/sqb.1956.021.01.026>.

Brandt, V. L., and D. B. Roth. 2008. “G.O.D.’s Holy Grail: Discovery of the RAG Proteins.” Journal of Immunology 180, no. 1: 3–4. <https://doi.org/10.4049/jimmunol.180.1.3>.

Burnet, F. M. 1959. The Clonal Selection Theory of Acquired Immunity. Cambridge University Press.

Burnet, F. M., and F. Fenner. 1949. The Production of Antibodies.2nd ed. Macmillan.

Burnet, F. M., M. Freeman, A. V. Jackson, and D. Lush. 1941. The Production of Antibodies, a Review and a Theoretical Discussion. Macmillan.

Cooper, M. D. 2010. “A Life of Adventure in Immunobiology.” Annual Review of Immunology 28: 1–19. <https://doi.org/10.1146/annurevimmunol-030409-101248>.

Crick, F. 1958. “On Protein Synthesis.” Symposia of the Society for Experimental Biology 12: 138–163.

Crick, F. 1989. “Neural Edelmanism.” Trends inNeuroscience (Tins) 12, no. 7: 240–248. <https://doi.org/10.1016/0166-2236(89)90019-2>.

Dubos, R. J. 1940. “The Adaptive Production of Enzymes by Bacteria.” *Bacteriological Reviews* 4, no. 1: 1–16. <https://doi.org/10.1128/br.4.1.1-16.1940>.

Edelman, G. M. 1987. *Neural Darwinism: The Theory of Neuronal Group Selection*. new ed. Basic Books.

Fagraeus, A. 1947. “Plasma Cellular Reaction and Its Relation to the Formation of Antibodies In Vitro.” *Nature* 159: 499.

Gruber, M. 1903. “Toxin Und Antitoxin: eine replik auf Herrn Ehrlichs entgegnung.” Wiener *KlinischeWochenschrift*, 16, 791–793.

Honjo, T. 2008. “A Memoir of AID, Which Engraves Antibody Memory on DNA.” *Nature Immunology* 9, no. 4: 335–337. <https://doi.org/10.1038/ni0408-335>.

Lindenmann, J. 1984. “Origin of the Terms ‘Antibody’ and ‘Antigen’.” *Scandinavian Journal of Immunology* 19, no. 4: 281–285. <https://doi.org/10.1111/j.1365-3083.1984.tb00931.x>.

Luria, S. E., and M. Delbruck. 1943. “Mutations of Bacteria From Virus Sensitivity to Virus Resistance.” *Genetics* 28: 491–511.

McDevitt, H. O. 2000. “Discovering the Role of the Major Histocompatibility Complex in the Immune Response.” *Annual Review of Immunology* 18: 1–17. <https://doi.org/10.1146/annurev.immunol.18.1.1>.

Neuberger, M. S. 2008. “Antibody Diversification by Somatic Mutation: From Burnet Onwards.” *Immunology and Cell Biology* 86: 124–132. <https://doi.org/10.1038/sj.icb.7100160>.

Nowell, P. C. 1976. “The Clonal Evolution of Tumor Cell Populations.” *Science* 194, no. 4260: 23–28. <https://doi.org/10.1126/science.959840>.

Prull, C. R. 2003. “Part of a Scientific Master Plan? Paul Ehrlich and the Origins of His Receptor Concept.” *Medical History* 47, no. 3: 332–356.

Reinherz, E. L. 2014. “Revisiting the Discovery of the Α*β* TCR Complex and Its Co-Receptors.” *Frontiers in immunology* 21, no. 5: 583. <https://doi.org/10.3389/fimmu.2014.00583>.

Talmage, D. W. 1957. “Allergy and Immunology.” *Annual Review of Medicine* 8: 239–256. <https://doi.org/10.1146/annurev.me.08.020157.001323>.

Vivier, E., and B. Malissen. 2005. “Innate and Adaptive Immunity: Specificities and Signaling Hierarchies Revisited.” *Nature Immunology* 6, no. 1: 17–21. <https://doi.org/10.1038/ni1153>.

Zinsser, H. 1914. *Infection and Resistance*. Macmillan.
